# A microcontroller-based system for flexible oxygen control in laboratory experiments

**DOI:** 10.1242/jeb.249207

**Published:** 2025-01-07

**Authors:** Stefan Mucha

**Affiliations:** Behavioral Physiology, Institute of Biology, Humboldt-Universität zu Berlin, 10099 Berlin, Germany

**Keywords:** Dissolved oxygen, Hypoxia, Acclimation, Respiratory physiology, Arduino, Environmental control

## Abstract

Environmental control systems are important tools for experimental researchers studying animal–environment interactions. Commercial systems for the measurement and regulation of environmental oxygen conditions are relatively expensive and cannot always be adapted to varying experimental applications. Here, I present a low-cost and highly flexible oxygen control system using Arduino microcontrollers in combination with a commercial optical oxygen sensor. Hardware and software examples are provided for three different applications: single-setpoint, sequential and long-term dissolved oxygen (DO) control. All tested control systems created the desired DO conditions with high accuracy and repeatability across trials. The resources provided shown here can be adapted and modified to be used in a variety of experimental contexts.

## Teaser

**Summary:** Development of a new, microcontroller-based oxygen control system for laboratory experiments.

## INTRODUCTION

Monitoring and regulation of environmental conditions are at the core of experimental studies investigating animal–environment interactions. An important environmental variable is oxygen availability. Almost all eukaryotes depend on oxygen for their survival (Fenchel, 2012). In most terrestrial habitats, oxygen is abundantly available (but see, for example, the naked mole rat or high-altitude habitats; Monge and León-Velarde, 1991; Pamenter, 2022). In aquatic habitats, dissolved oxygen (DO) can fluctuate significantly on a small spatial and temporal scale (Kramer, 1984; Graham, 1990). In addition to naturally occurring conditions of low DO (hypoxia), global warming and environmental degradation (e.g. eutrophication) are projected to lead to an increased frequency and intensity of hypoxic events (Diaz and Rosenberg, 2008; McBryan et al., 2013; Breitburg et al., 2018). Many experimental studies test how animals respond to changes in environmental oxygen by exposing them to acute hypoxic stress. Such experiments are often combined with chronic oxygen treatments to control for longer-term effects of DO environment (Prosser et al., 1957; Lomholt and Johansen, 1979; Love and Rees, 2002; Shang et al., 2006; Reardon and Chapman, 2010; Cook et al., 2013; Ding et al., 2013; Borowiec et al., 2015, 2018, 2020; Collins et al., 2015; Pan et al., 2017; Ackerly et al., 2018, 2023).

To establish short-term and long-term DO regimes, automated measurement and control systems have many benefits over manual manipulation of environmental conditions: they create reproducible and stable conditions, they operate continuously over extended periods and they save time and labor for researchers.

Establishing automated DO control in a laboratory is not trivial. The efficiency of DO control depends on many variables that should be taken into consideration, such as water volume, gas pressure and mode of delivery, surface area for exchange with atmospheric oxygen, temperature, water flow and mixing, and desired DO concentration. And because of weathering of components, no automated control system can work without regular maintenance and control of an experimenter. Various manufacturers offer off-the-shelf environmental control systems. While these systems have the benefit of professional support and service, they can easily cost several tens of thousands of US dollars and, once established, are not always adaptable for a different desired experimental design (e.g. establishing constant conditions versus fluctuating DO, setting stable conditions versus defined change rates, interfacing with a PC versus standalone logging and control). The inaccessibility of DO control systems limits ecophysiological research, as it allows only a few, well-funded laboratories to conduct studies with controlled DO regimes.

Recently, Ern and Jutfelt (2024) addressed this issue by publishing an inexpensive do-it-yourself oxygen control system (OptoReg). In this article, an alternative framework for automated DO control using Arduino microcontroller boards (https://www.arduino.cc/) is presented. Both the microcontroller-based system described here and the OptoReg use the same oxygen meter (FireStingO2, PyroScience GmbH, Aachen, Germany), but there are some important differences. The OptoReg system exploits the meter's function to generate an analog output signal when a predefined threshold is crossed, making it well suited for experiments with static DO setpoints. In contrast, the microcontroller-based system shown here uses a serial connection to interface with the sensor, giving it full control of the meter's functions. The system can be programmed to create a range of DO regimes for static and dynamic experiments with controlled change rates. It supports a variety of output devices (solenoid valves, motors, relays) and can be connected to a computer or used as stand-alone logging and control unit, which can be useful in fish holding facilities or field settings.

The aim of this article is to provide researchers with the knowledge and tools necessary to realize their own microcontroller-based DO control system. It gives a brief introduction to the operating principles of microcontrollers and shows different hardware and software configurations for acute and long-term hypoxia exposure experiments.

## MATERIALS AND METHODS

A full list of components and manufacturers can be found in Table 1. In all experiments, DO was controlled by regulating the flow of air or nitrogen gas (Alphagaz 1, Air Liquide S.A., Paris, France) into the sample at a maximum gas pressure of 1 bar (100 kPa). R version 4.0.4 (https://www.r-project.org/) was used for data analysis. Figures were formatted with Adobe Illustrator (Adobe Inc., San Jose, CA, USA). Serial logger software (ExtraPuTTY) was used to display and log data from the control system on a computer. All data and R scripts are available from figshare (https://doi.org/10.6084/m9.figshare.25764879.v3).

**Table 1.** Components of the oxygen control systems

Reference	Component	Model	Price (USD)
All examples	DO meter	FireStingO2 (FSO2-C4), PyroScience GmbH, Aachen, Germany	4480
	DO sensor	Robust Oxygen Probe (OXROB10), PyroScience	380
	Temperature sensor	Pt100 Temperature Probe (TSUB21), PyroScience	180
	7-Pin connector	PTSM 0,5/ 7-P-2,5, art. no. 1778887, Phoenix Contact, Nanjing, China	4
	Cables and supplies	Generic	20
Gas delivery	Gas diffusor	Aquarium Nano Bubble Stone 60 mm, Boxtech, Shenzen, China	14
	Gas tubing	Generic (6 mm outer diameter pneumatic hose)	–
	Nitrogen	Alphagaz 1, Air Liquide, Paris, France	–
Examples 1 and 2	Microcontroller	Arduino Uno, Arduino, Monza, Italy	25
	Relay board	TC-9072472 2 Relay Module, Tru Components, Wels, Austria	8
	Solenoid valve	Pro Valve 621.06.04131.12VDC, Provalve Armaturen, Hohe Börde, Germany	40
	Valve adapter plug	Precon B-12443-0127 12 - 24V/DC, Nass Magnet, Hanover, Germany	10
	12 V power supply	Generic (VC-11320935, Voltcraft, Düsseldorf, Germany)	15
	Water pump	Generic (CompactON300, Eheim, Deizisau, Germany)	20
	Heating rod	Generic (Thermocool 200, Eheim)	25
	Thermostat	ITC-308, Inkbird, Shenzhen, China	30
Example 3	Microcontroller	Arduino Mega 2560, Arduino	50
	Display	LCD Shield kit, Adafruit Industries, New York, NY, USA	25
	SD card logging	Data Logger Shield, Adafruit Industries	15
	4-Relay board	TC-9927216, Tru Components	13
	4 Solenoid valves	6011 24V (art. 163521), Bürkert, Ingelfingen, Deutschland	240
	5 V power supply	Generic (VC-11258705, Voltcraft)	20
	9 V power supply	Generic (VC-11258705, Voltcraft)	20
	24 V power supply	Generic (OWA-60E-24, Mean Well, New Taipei City, Taiwan)	30
	Housing	Generic (2007125K, OBO Bettermann, Menden, Germany)	30

Prices are estimated based on a survey of online shops (as of May 2024). See Table S1 for a version of this table with additional supplier information.

### Choice of hardware components

The control systems shown here were designed to be used with the optical FireStingO2 meter (PyroScience GmbH). This meter was chosen because it is easy to connect to a microcontroller (e.g. Ern and Jutfelt, 2024) and its communication protocol is available on the manufacturer's website (https://www.pyroscience.com/). The sensors were calibrated according to the manufacturer's instructions, using the supplied software.

Arduino boards (Arduino LLC, Monza, Italy) were used as control units because they are inexpensive, comparatively easy to program, and offer a large number of software and hardware add-ons (libraries and shields). Arduino boards are designed to execute user-written programs (sketches), which are stored on the microcontroller's flash memory and executed when the device is powered up.

To regulate gas flow, different components were tested: (1) solenoid valves; (2) mass-flow controllers; and (3) motorized needle valves. The setups shown here use solenoid valves because they are commonly available, relatively inexpensive and easy to use. Examples of systems with mass-flow controllers and motorized needle valves are included in the Supplementary Materials and Methods (Examples S2 and S3) and Figs S2 and S3.

### Connecting the microcontroller and oxygen meter

**Fig. 1.**
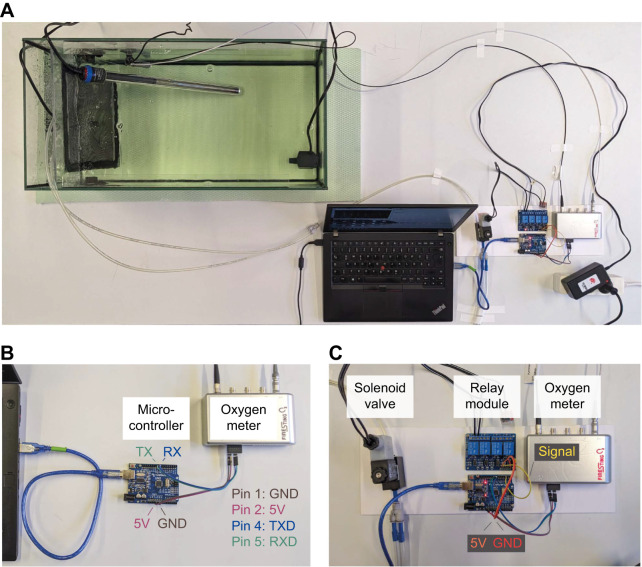
**Example setup for microcontroller-based DO control.** (A) Top-down overview. Nitrogen gas is diffused into the left side of the tank via two gas diffusors. A barrier with black plastic mesh separates the gas diffusors from the rest of the tank. Two small pumps are used to mix the water in the tank and a heating rod, connected to a thermostat, is placed in the tank to maintain a steady temperature. A temperature probe and a robust dissolved oxygen (DO) probe are placed in the tank and connected to the FireStingO2 meter. (B) The Arduino is connected to a computer via USB and to the X1-connector of the oxygen meter using jumper wires. (C) The ground terminal of the power supply for the solenoid valve is routed through the normally open contacts of a relay module. GND, ground; RX/RXD, receive-pin; TX/TXD, transfer-pin.

The FireStingO2 meter was connected to an Arduino Uno microcontroller via a Universal Asynchronous Receiver Transmitter (UART) serial connection (Fig. 1B). To connect the devices, jumper wires and a 7-pin PCB connector were used (Table 1). For data transfer, two digital pins of the Arduino (pins 8 and 9) were connected to pins 4 and 5 on the meter's X1 connector, which is a 7-pin connector located at the back side of the device, respectively. The meter was powered by connecting the microcontroller's ground (GND) and positive (5V) pins to the meter's pins 1 and 2, respectively. This ensured that both devices operated on the same electrical ground, which is required for the serial communication to work (see Fig. S1 and Supplementary Materials and Methods Example S1 for more detailed instructions on setting up serial communication between microcontrollers, sensors and computers).

### Controlling nitrogen flow

Nitrogen flow was controlled by solenoid valves whose power supply was switched on and off via a relay module operated by the Arduino. The Arduino's 5V, GND and digital pins were connected to the relay module's 5V, GND and input pins, respectively, using jumper wires (Fig. 1C). The power supply's positive terminal was connected directly to the positive contact of the valve, and the GND terminal was routed through the normally open contacts of the relay boards to the GND contact of the valve. A flyback diode was connected between the valve contacts with forward direction from the GND to positive contact. This diode is required to short-circuit voltage spikes that result from inductive loading of the solenoid coil during operation. An adapter plug was used to facilitate the connections to the valve contacts, which had an integrated flyback diode. When the relay module received a digital ‘on’ signal (HIGH), it closed the power circuit of the valve, thus allowing nitrogen gas to flow into the sample.

### Programming the microcontroller and transferring data

**Fig. 2.**
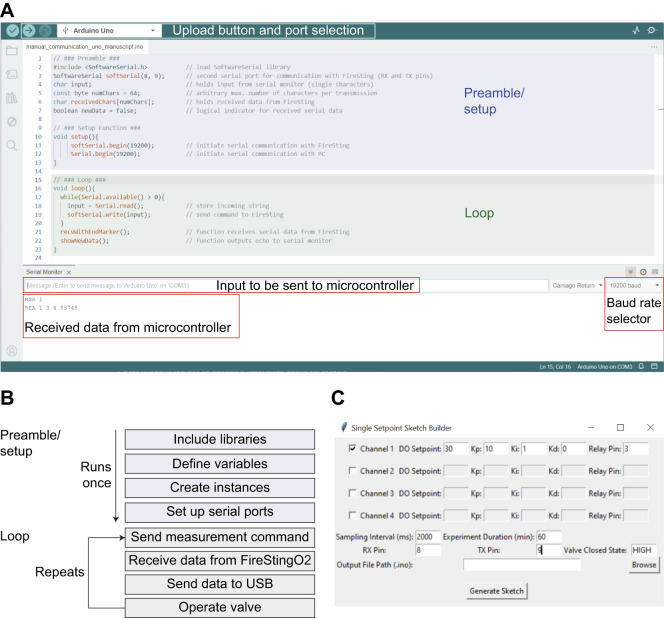
**Software for programming Arduino microcontrollers and DO control.** (A) Screenshot of the Arduino IDE main window. Code sections are marked with colors corresponding to the sections in B. (B) Example program flow of a DO control sketch. (C) Screenshot of the Python Sketch Builder window. See Materials and Methods for explanations.

DO control sketches were written in the Arduino programming language and uploaded to the microcontroller via the Arduino Integrated Development Environment (IDE, https://www.arduino.cc/en/software, v.2.3.3; Fig. 2A). All Arduino sketches shown here are included in the ‘Ardoxy’ software library, which can be downloaded from GitHub (https://github.com/muchaste/ArdOxy) and integrated into the IDE by copying the ‘Ardoxy’ folder of the GitHub repository into the ‘libraries’ directory of the IDE installation. Example sketches can be accessed by clicking File>Examples>Ardoxy in the main application window.

To upload sketches, the board is connected to a computer via USB, and the corresponding sketch is opened in the IDE. After selecting the COM port and model of the connected board, the sketch is uploaded by clicking the ‘Upload’ button (Fig. 2A).

Arduino sketches consist of two main sections: (1) the preamble and setup function, where libraries are included, variables and instances are declared, and the board is configured; and (2) the main loop, which is executed repeatedly as long as the Arduino is running or until a stop condition is met (Fig. 2B). Once the sketch is uploaded, the microcontroller executes it when powered up. Depending on programming, microcontrollers can send data to a computer (e.g. examples 1 and 2) or function as standalone units (e.g. example 3).

Except for the standalone DO control system, all example programs were designed for the microcontroller to send measured values to the computer and be controlled by commands sent from the computer via a serial terminal software. The Arduino IDE offers a simple serial monitor function (Fig. 2A), which lacks the capability to record serial data. For data logging on the computer, ExtraPuTTY (https://sourceforge.net/projects/extraputty/) was used, as it can be configured to save serial data in a text file for later analysis. A variety of serial terminal programs are freely available and can be chosen based on compatibility and functionality (e.g. serialplot, https://github.com/hyOzd/serialplot, which offers real-time plotting and logging of serial data). To set up data transfer, the correct COM port has to be selected and the rate of data exchange (baud rate) must be set to match between sender and receiver. All examples provided here use a baud rate of 19,200 pulses s^−1^.

### Example 1: DO control in a static experiment

In this experiment, DO was regulated in a rectangular aquarium (60×30×35 cm, 0.18 m² surface area, 47 l water volume) from normoxic values (>99% air saturation) to 30% air saturation for 1 h. Two small pumps were used to mix the water in the tank and a heating rod, connected to a thermostat, was placed in the tank to maintain a temperature of 25°C. A temperature probe and a robust DO probe were placed in the tank and connected to the FireStingO2 meter. The Arduino was connected to a computer via USB. The components of the control system (relay, valve, board and meter) were secured on a plastic plate (Fig. 1A, Table 1).

A control sketch was written to measure DO and temperature, send the values to the computer via serial connection, and operate the valve (Fig. 1B). The sketch can be found in the ‘Ardoxy’ library (https://github.com/muchaste/ArdOxy) under the example ‘setpoint_solenoid’ and users can adapt it to their specific experimental needs using the Arduino IDE. Alternatively, a Python program is included in the GitHub repository of the library, ‘Single Setpoint Sketch Builder’, which provides a graphical user interface (GUI) for creating similar Arduino sketches for static DO control on up to four channels (Fig. 2C). A list of parameters, their corresponding variable name in the sketch, and example values are provided in Table 2. The opening time of the valve is computed using proportional-integral-derivative (PID) control algorithms from the PID library (https://github.com/br3ttb/Arduino-PID-Library). In this example, the weights for the PID control parameters (Kp, proportional; Ki, integral; Kd, derivative) were set for a dominant proportional (Kp=10), small integral (Ki=1) and zero differential (Kd=0) component. The sample interval was set at 2000 ms. The meter was connected to the digital pins 8 and 9 on the Arduino, and the relay, which operated the valve, was connected to digital pin 3. The relay pin state, at which the valve is closed, can be either HIGH or LOW, depending on the wiring and type of valve and relay. Here, the valve was closed when the pin was set to HIGH (this setting can be easily determined by trial and error).

**Table 2.** Parameters, variable names and example values for static DO control

Parameter	Variable name in sketch	Example value
Channel number	channelNumber	1
DO setpoint (% air saturation)	DOSetpoints	30
Kp	Kp	10
Ki	Ki	1
Kd	Kd	0
Sampling interval (ms)	sampInterval	2000
Experiment duration (min)	experimentDuration	60
RX pin number	Rx	8
TX pin number	Tx	9
Pin number for relay operation	relayPins	3
Valve closed state	closed	HIGH

### Example 2: DO control with multiple setpoints and controlled rates of change

In this experiment, a similar setup as in example 1 was used. The control sketch was modified to regulate DO values to a sequence of setpoints, from normoxia to 50% and then to 30% air saturation, with a change phase and holding phase of 15 min each. The sketch can be found in the ‘Ardoxy’ library (https://github.com/muchaste/ArdOxy) under the example ‘sequence_solenoid’. In addition to the parameters from the previous experiment (Table 2), users have to define the number of phases (i.e. each step of the sequence, here: 4), and the durations and regulation types (‘c’ for change, ‘h’ for hold) for each phase in the preamble of the sketch. After startup, the microcontroller automatically generates a list of time points for the transition from each phase to the next. By comparing the current time with these time points, the microcontroller determines which setpoint to use and which control algorithm to execute. A second PID instance is used for control during the change phases, which can be tuned with its own set of parameter weights. During the change phases, the system controls the DO change rate rather than absolute values. To avoid drift during the change phases, the change rate is recalculated at regular intervals. Currently, this program only supports one control channel.

### Example 3: long-term standalone DO control

In this experiment, long-term hypoxic conditions were established in four rectangular aquariums (80×30×35 cm, 0.24 m² surface area, 70 l water volume) without the need for a connected computer. The tanks were equipped with one pump and one gas diffusor each, and bubble wrap was placed on the water surface to reduce diffusion of oxygen from the ambient air into the water. DO levels were decreased from normoxia to 70% air saturation on the first day, and then reduced in steps of 10% air saturation per day. On the final day, DO was reduced by 5% to reach 15% air saturation, which was maintained for 8 weeks.

An Arduino Mega 2560 was used because of its larger memory and additional connection pins compared with the Arduino Uno, which makes it theoretically possible to scale up the control system. The board was connected to one FireStingO2 meter as described above, with a robust DO probe placed approximately in the middle of each tank and the temperature probe in a randomly selected tank. DO and temperature were measured every 60 s, displayed on an LCD, and logged on an SD card using a datalogger shield. Nitrogen flow was controlled using a 4-relay module and four 24 V solenoid valves. The components of the control system were housed in a waterproof wall mount box (Table 1). All tanks were kept in a temperature-controlled room to ensure approximately similar conditions across tanks (average temperature: 25.4±0.4°C).

The control sketch for this long-term experiment was largely similar to the previous examples. Because of the long duration of this experiment, it uses time data from the real-time clock of the datalogger shield. The user has to provide the starting date for DO regulation, the sequence of DO setpoints and the duration for maintaining each setpoint. Based on this information, the program keeps track of the corresponding setpoint throughout the experiment. As the real-time clock is battery powered and the sketch is stored in the Arduino's memory, the system can resume regulation after brief power outages.

## RESULTS AND DISCUSSION

This study shows how to assemble DO control systems using inexpensive and open source components. Hardware and software examples are provided for three different applications: single-setpoint, sequential and long-term DO control. All tested control systems created the desired DO conditions with an accuracy that is comparable to that of other systems (Table S4), and with a high repeatability across trials. The examples shown here can be adapted and modified to be used in a variety of experimental contexts (see also Supplementary Materials and Methods Examples 1–3 and Figs S1–S3).

### Example 1: DO control in a static experiment

**Fig. 3.**
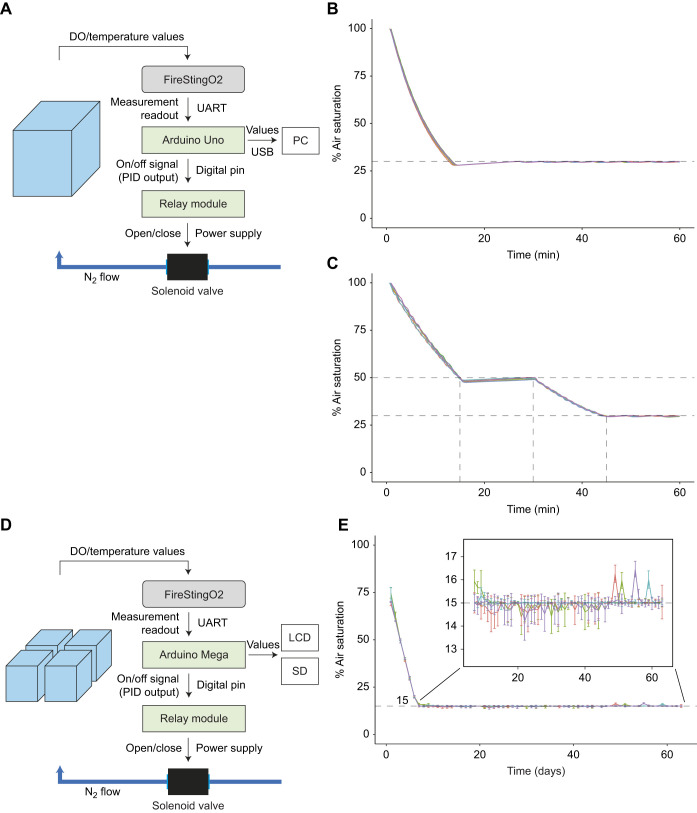
**Schematic representation of control systems (left) and corresponding results (right) for the three experimental setups.** (A–C) Diagram of the short-term DO control system (A) and the acute DO changes with single setpoints (static, *n*=6 trials; B) and multiple setpoints with fixed rates (sequence, *n*=6 trials; C). (D,E) Long-term DO control system (D) with control across four tanks and the daily DO (means and standard deviation, *n*=4; E). Dashed horizontal lines represent target DO setpoints, and dashed vertical lines mark transitions between phases of the sequence. PID, proportional-integral-derivative controller.

The control system lowered air saturation to below 30% within 15 min and generated a slight undershoot, which evened out within 30 min as a result of passive diffusion of oxygen into the water from the air. After the initial undershoot, the system maintained DO at 29.88±0.19% air saturation. Repeatability across trials was high (*n*=6 trials, Pearson's *R*≥0.999; Fig. 3A,B).

### Example 2: DO control with multiple setpoints and controlled rates of change

The first setpoint at 50% air saturation was reached within 15 min with a slight undershoot, which evened out over the 15 min of the holding phase as a result of passive diffusion of oxygen into the water from the air (48.88±0.65% air saturation during the holding phase). The second setpoint at 30% air saturation was reached within 15 min and was maintained at a higher accuracy (29.72±0.28% air saturation). Repeatability across trials was high (*n*=6 trials, Pearson's *R*≥0.999; Fig. 3C).

### Example 3: long-term standalone DO control

Long-term control created the desired DO conditions over 8 weeks quite reliably (14.96±0.38% air saturation) and DO values were highly correlated across tanks (Pearson's *R*≥0.999, *n*=4; Fig. 3D,E).

### Optimization, advantages and limitations

The accuracy of DO control depends on many parameters that are not controlled by the system, such as gas pressure, bubble size and water mixing (see ‘Technical considerations’, below). However, certain settings in the control algorithms can be adjusted to improve the performance of the control system. In Example 1, the fast reductions in DO resulted in temporary undershoots, which were substantially reduced in Example 2, where the rate of DO change was controlled and DO was changed in a stepwise manner. Thus, using slower change rates may improve DO control accuracy.

In some cases, fine-tuning the PID controller can further improve accuracy. This is done by adjusting the weights for the main parameters (Kp, Ki, Kd). Based on experience, giving the highest weight to proportional control while keeping integral and derivative control values low or zero will be sufficient for accurate DO regulation. It is recommended that preliminary tests are run to determine whether fine-tuning of the PID controller yields improvements. There are numerous resources available to help find the best values for these parameters, both in the literature (e.g. Borase et al., 2021) and online (e.g. https://tlk-energy.de/blog-en/practical-pid-tuning-guide; https://pidexplained.com/how-to-tune-a-pid-controller/).

One major advantage of using microcontrollers is their flexibility. They can control virtually any valve type and establish a variety of oxygen regimes (see Supplementary Materials and Methods Example 3, Table S3 and Fig. S3 for stepwise DO change in a shuttle-box). To make the best use of the features that microcontrollers offer, and to ensure proper working of the system, researchers should acquire some knowledge of the Arduino programming language and functioning principle. Choosing and assembling the hardware are up to the experimenter and will require time and hands-on learning. The instructions, hardware lists (Table 1 and Table S1), code examples and GUI tool provided here should help to facilitate the first steps for the most common use cases.

At present, the ‘Ardoxy’ library is compatible only with the FireStingO2 meter. Integration of other meters and sensors is possible, provided they can be connected to a microcontroller and that their communication protocol is known in the case of a serial connection. Optical DO sensors are beneficial in automated DO control systems because, in contrast to electrochemical sensors, they show little drift over time and do not consume oxygen during measurement. However, in applications where electrochemical sensors are suitable (e.g. short-term experiments), they may be considered as an alternative because of their low price. Commercial kits are available that convert DO readings from an electrochemical probe into analog voltage values that can be read out by an Arduino using the analog input pins (see Table S1). By replacing the FireStingO2-specific code sections in the examples shown here with code to read values from these sensors, they could be integrated whilst maintaining the other functionalities of the control programs (such as computation of control outputs, operation of valves).

Calibration of the oxygen meter is not included in the ‘Ardoxy’ library and must be done using the manufacturer's software. Additionally, the communication protocol of the meter varies depending on the firmware version. The functions in the ‘Ardoxy’ library are designed to automatically detect the firmware version and send the correct measurement and readout commands for firmware versions 3–4. To anticipate future changes in the communication protocol, I have also included generic versions of the measurement and readout functions. These functions accept character strings as arguments, allowing users to manually input commands if the preset commands are incompatible with future firmware updates (see Table S2).

### Technical considerations

In experimental settings that create a high latency between control output (nitrogen pulse) and effect (DO reduction), it is difficult to achieve accurate DO control. While this issue cannot be fully resolved in most cases, there are some simple measures that experimenters can take in order to minimize latency: ensure good water mixing; minimize compartmentalization; use the shortest possible measurement interval that does not lead to oversampling.

The water volume and surface area also play a role and should be considered if possible. When DO levels change dynamically (e.g. during an acute hypoxia challenge), using a small water volume can reduce the latency of DO regulation. When DO levels should remain static over a longer time, using larger water volumes can be beneficial as they are more stable in their DO content. Covering the water surface directly, e.g. with bubble wrap, significantly reduces the diffusion of oxygen into the water, which reduces the usage of nitrogen gas and facilitates the maintenance of static DO conditions (Fig. S4).

Over longer experimental durations, the biggest challenge may not be the physical process of DO regulation but rather the maintenance of the system. Ensuring a reliable supply of nitrogen gas (without pressure fluctuations), as well as the functioning of all components of the system throughout the experimental duration are obvious minimum requirements for reliable control of DO. During the 9 weeks of the long-term DO control shown here, I encountered weathering of the DO probes, a malfunctioning solenoid valve and a supply shortage of bottled nitrogen gas. Most of these factors can be anticipated by keeping a contingency of spare components and backup nitrogen gas, as well as conducting regular maintenance.

### Conclusion

This study shows that microcontrollers are well suited to control DO conditions both in short-term and long-term applications. As control algorithms for environmental control systems are largely similar across parameters, the modular nature of microcontroller-based systems makes it possible to extend this control system to other parameters, such as temperature (e.g. by exchanging the DO sensor with a temperature probe and the solenoid valve with a heating rod). Together with the recently published OptoReg System (Ern and Jutfelt, 2024), microcontroller-based systems enhance the spectrum of inexpensive do-it-yourself methodology for environmental control systems. Integrating these systems requires researchers to engage with, and understand, the nuts and bolts of their experimental setups and facilitates sharing of code and data, supporting open and reproducible science.

## Supplementary Material

10.1242/jexbio.249207_sup1 Supplementary information

## References

[JEB249207C1] Ackerly, K. L., Krahe, R., Sanford, C. P. and Chapman, L. J. (2018). Effects of hypoxia on swimming and sensing in a weakly electric fish. *J. Exp. Biol.* 221, jeb172130. 10.1242/jeb.172130 30018158

[JEB249207C2] Ackerly, K. L., Negrete, B., Dichiera, A. M. and Esbaugh, A. J. (2023). Hypoxia acclimation improves mitochondrial efficiency in the aerobic swimming muscle of red drum (*Sciaenops ocellatus*). *Comp. Biochem. Physiol. A Mol. Integr. Physiol.* 282, 111443. 10.1016/j.cbpa.2023.111443 37201653

[JEB249207C3] Borase, R. P., Maghade, D. K., Sondkar, S. Y. and Pawar, S. N. (2021). A review of PID control, tuning methods and applications. *Int. J. Dynam. Control* 9, 818-827. 10.1007/s40435-020-00665-4

[JEB249207C4] Borowiec, B. G., Darcy, K. L., Gillette, D. M. and Scott, G. R. (2015). Distinct physiological strategies are used to cope with constant hypoxia and intermittent hypoxia in killifish (*Fundulus heteroclitus*). *J. Exp. Biol.* 218, 1198-1211. 10.1242/jeb.114579 25722002

[JEB249207C5] Borowiec, B. G., McClelland, G. B., Rees, B. B. and Scott, G. R. (2018). Distinct metabolic adjustments arise from acclimation to constant hypoxia and intermittent hypoxia in estuarine killifish (*Fundulus heteroclitus*). *J. Exp. Biol.* 221, jeb190900. 10.1242/jeb.190900 30518600

[JEB249207C6] Borowiec, B. G., Hoffman, R. D., Hess, C. D., Galvez, F. and Scott, G. R. (2020). Interspecific variation in hypoxia tolerance and hypoxia acclimation responses in killifish from the family Fundulidae. *J. Exp. Biol.* 223, jeb209692. 10.1242/jeb.209692 31988166 PMC7044458

[JEB249207C7] Breitburg, D. L., Levin, L. A., Oschlies, A., Grégoire, M., Chavez, F. P., Conley, D. J., Garçon, V., Gilbert, D., Gutiérrez, D., Isensee, K. et al. (2018). Declining oxygen in the global ocean and coastal waters. *Science* 359, eaam7240. 10.1126/science.aam7240 29301986

[JEB249207C8] Collins, G. M., Clark, T. D. and Carton, A. G. (2015). Physiological plasticity v. inter-population variability: understanding drivers of hypoxia tolerance in a tropical estuarine fish. *Mar. Freshw. Res.* 67, 1575-1582. 10.1071/MF15046

[JEB249207C9] Cook, D. G., Iftikar, F. I., Baker, D. W., Hickey, A. J. R. and Herbert, N. A. (2013). Low-O_2_ acclimation shifts the hypoxia avoidance behaviour of snapper (*Pagrus auratus*) with only subtle changes in aerobic and anaerobic function. *J. Exp. Biol.* 216, 369-378. 10.1242/jeb.073023 23038727

[JEB249207C10] Diaz, R. J. and Rosenberg, R. (2008). Spreading dead zones and consequences for marine ecosystems. *Science* 321, 926-929. 10.1126/science.1156401 18703733

[JEB249207C11] Ding, Z., Sun, P., Hua, X., Bai, Y., Shang, E. H. H., Wu, R. S. S. and Zuo, Y. (2013). mRNA expression of select hypoxia-inducible genes and apoptotic control genes in zebrafish exposed to hypoxia during development. *Pol. J. Environ. Stud.* 22, 357-365.

[JEB249207C12] Ern, R. and Jutfelt, F. (2024). The OptoReg system: a simple and inexpensive solution for regulating water oxygen. *Conserv. Physiol.* 12, coae024. 10.1093/conphys/coae024 38737128 PMC11087874

[JEB249207C13] Fenchel, T. (2012). Anaerobic eukaryotes. In *Anoxia*, Vol. 21 (ed. A. V. Altenbach, J. M. Bernhard and J. Seckbach), pp. 3-16. Dordrecht: Springer.

[JEB249207C14] Graham, J. B. (1990). Ecological, evolutionary, and physical factors influencing aquatic animal respiration. *Am. Zool.* 30, 137-146. 10.1093/icb/30.1.137

[JEB249207C15] Kramer, D. L. (1984). The evolutionary ecology of respiratory mode in fishes: an analysis based on the costs of breathing. In *Evolutionary Ecology of Neotropical Freshwater Fishes*, Vol. 3 (ed. E. K. Balon and T. M. Zaret), pp. 67-80. Dordrecht: Springer.

[JEB249207C16] Lomholt, J. P. and Johansen, K. (1979). Hypoxia acclimation in carp - how it affects O_2_ uptake, ventilation, and O_2_ extraction from water. *Physiol. Zool.* 52, 38-49. 10.1086/physzool.52.1.30159930

[JEB249207C17] Love, J. W. and Rees, B. B. (2002). Seasonal differences in hypoxia tolerance in gulf killifish, *Fundulus grandis* (Fundulidae). *Environ. Biol. Fish.* 63, 103-115. 10.1023/A:1013834803665

[JEB249207C18] McBryan, T. L., Anttila, K., Healy, T. M. and Schulte, P. M. (2013). Responses to temperature and hypoxia as interacting stressors in fish: implications for adaptation to environmental change. *Integr. Comp. Biol.* 53, 648-659. 10.1093/icb/ict066 23784697

[JEB249207C19] Monge, C. and León-Velarde, F. (1991). Physiological adaptation to high altitude: oxygen transport in mammals and birds. *Physiol. Rev.* 71, 1135-1172. 10.1152/physrev.1991.71.4.1135 1924550

[JEB249207C20] Pamenter, M. E. (2022). Adaptations to a hypoxic lifestyle in naked mole-rats. *J. Exp. Biol.* 225, jeb196725. 10.1242/jeb.196725 35188211

[JEB249207C21] Pan, Y. K., Ern, R., Morrison, P. R., Brauner, C. J. and Esbaugh, A. J. (2017). Acclimation to prolonged hypoxia alters hemoglobin isoform expression and increases hemoglobin oxygen affinity and aerobic performance in a marine fish. *Sci. Rep.* 7, 7834. 10.1038/s41598-017-07696-6 28798467 PMC5552867

[JEB249207C22] Prosser, C. L., Barr, L. M., Pinc, R. D. and Lauer, C. Y. (1957). Acclimation of goldfish to low concentrations of oxygen. *Physiol. Zool.* 30, 137-141. 10.1086/physzool.30.2.30155362

[JEB249207C23] Reardon, E. E. and Chapman, L. J. (2010). Hypoxia and energetics of mouth brooding: is parental care a costly affair? *Comp. Biochem. Physiol. A Mol. Integr. Physiol.* 156, 400-406. 10.1016/j.cbpa.2010.03.007 20227513

[JEB249207C24] Shang, E. H. H., Yu, R. M. K. and Wu, R. S. S. (2006). Hypoxia affects sex differentiation and development, leading to a male-dominated population in zebrafish (Danio rerio). *Environ. Sci. Technol.* 40, 3118-3122. 10.1021/es0522579 16719120

