# Supplementary material for: A microcontroller-based system for flexible oxygen control in laboratory experiments
Source: J Exp Biol. 2025 Jan 7;228(1):jeb249207. doi: 10.1242/jeb.249207 (PMC11744317; doi:10.1242/jeb.249207)
Supplement: Supplementary information [file jexbio-228-249207-s1.pdf]

Supplementary Materials and Methods

Example S1: Communicating with the FireStingO$_2$ via an Arduino

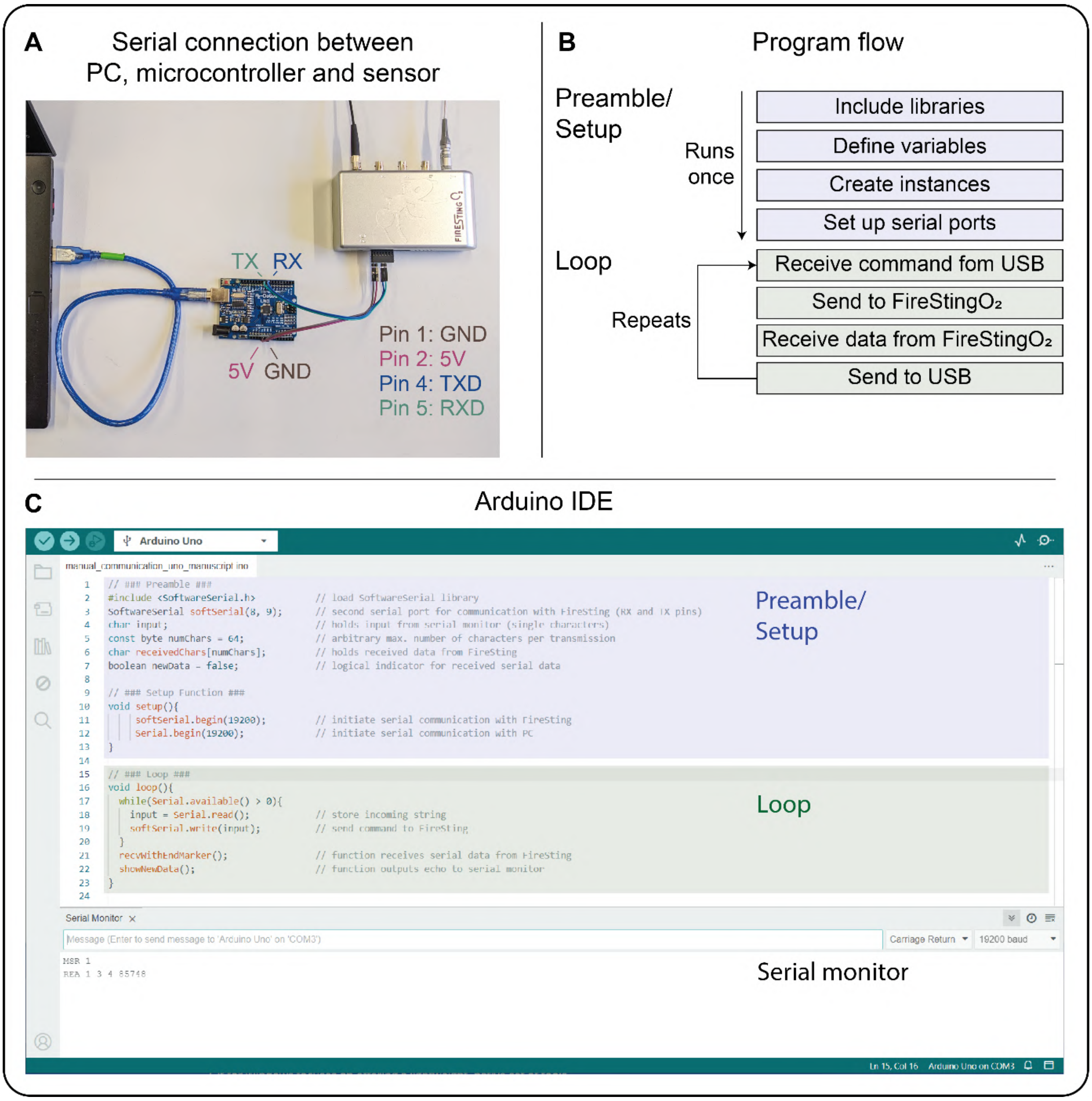

Fig. S1. Minimal example for serial communication between computer, microcontroller, and DO meter. A: Wiring example for connecting an Arduino Uno-type microcontroller to a computer and the FireStingO2 meter via serial connection. Note that the receive-pin (RX) of the microcontroller is connected to the transfer-pin (TXD) of the meter and vice versa. B: Program flow of the Arduino sketch for relaying serial data between the meter and the computer. First, preamble and setup function are executed once, followed by a program loop that is repeated while the Arduino is running. C: Screenshot of the Arduino IDE. Code sections are marked with colors corresponding to the sections in B. The function 'recvWithEndMarker()' stores the serial data from the meter in a character array ('receivedChars' in the preamble), and 'showNewData()' sends it to the computer. GND: ground, PID: proportional-integral-differential controller, RX/RXD: receive-pin, TX/TXD: transfer-pin.

Journal of Experimental Biology • Supplementary information

This is a minimal example for setting up communication between FireStingO_2 and Arduino. The FireStingO_2 meter was connected to the Arduino microcontroller via a Universal Asynchronous Receiver Transmitter (UART) – type serial connection (Fig. S1A, Table S1). Importantly, both devices have to operate on the same electrical ground for the serial communication to work. This can be achieved by powering the FireStingO_2 directly from the microcontroller's 5V/GND-pins. To connect the devices, jumper wires and a 7-pin PCB connector were used. The Arduino was connected to a computer via USB and a temperature probe and a robust DO probe were connected to the meter. The sketch shown is included as example in the 'Ardoxy' library ('manual_communication_uno'). Arduino sketches consist of two sections - the preamble and setup function, where libraries are included, variables and instances are declared, and the board is configured; and the main loop, which is executed repeatedly as long as the Arduino is running or until a stop condition is met (Fig. S1B). In the preamble and setup section of the example sketch, two serial connections are set up – one between PC and Arduino, and one between Arduino and FireStingO_2 (Fig. S1B-C). Since the Arduino Uno R3 offers by default only one serial port, the 'SoftwareSerial' library is included to create an additional port. The rate of data exchange (baud rate) is passed as argument to the functions initiating the serial connections ('begin()'), and must match between sender and receiver. The baud rate of the meter can be found in the manual, and the baud rate of the computer can be set in the Arduino IDE (Fig. S1C, serial monitor, top right corner). Here, all devices use a baud rate of 19200 pulses/s. The functions within the loop section relay serial data between the computer and the meter. Commands to the meter are input by the user via the serial monitor of the Arduino IDE and sent with a 'Carriage Return' as end marker. If the meter recognizes the command (i.e., if it matches one of the commands defined in the manufacturer's communication protocol), it returns an echo of the command, and, in case of a readout request, the corresponding value. In this example, two commands were sent to the meter and echoed: "MSR 1" to trigger a DO-measurement on channel 1, and "REA 1 3 4" to return the measurement result. The echo of the last command is followed by the DO value (in percent air saturation x 1000). In this case, an air saturation of 85.748% was measured (Fig. S1C). The commands are defined in the communication protocol of the FireStingO_2, which depends on the firmware version of the device and is supplied by the manufacturer.

Journal of Experimental Biology • Supplementary information

Example S2: DO Control with Fixed DO Change Rates using a Motorized Needle Valve

Using a motorized needle valve creates a continuous gas flow, which can potentially increase the accuracy of DO regulation and reduce the disturbance of the fish through nitrogen gassing. In systems using solenoids, disturbances through sudden intervals of nitrogen gassing can occur and may make the use of sumps or header tanks necessary, in which the gas is diffused into the water and which are connected to the tank housing the fish. In this experiment, we changed DO in rectangular fish tanks (80x30x35cm, water volume of 70L) from normoxic values (>90% air saturation) to 10% air saturation and back to 95% air saturation at a defined change rate (Fig. S2). During the trials, water in the tank was mixed with a small pump (CompactON300, Eheim, Deizisau, Germany) and bubble wrap was placed on the water surface to reduce the diffusion of atmospheric oxygen into the water. The DO control system consisted of an Arduino Uno R3 that was connected to a FireStingO_2 sensor via serial connection (Fig. S1, S2A). A temperature probe (TSUB-21, PyroScience) and a robust DO probe (OXROB 10-CL4, PyroScience) were placed approximately in the middle of the tank. Temperature and DO were measured every two seconds, values were sent from the Arduino to a PC via USB connection and visualized using SerialPlot (https://github.com/hyOzd/serialplot, Fig. S2B). The gas flow was regulated through a needle valve that was connected to a stepper motor via a shaft coupler. A motor shield (Motorshield V2, Adafruit, New York, USA) was used to drive the motor via the Arduino. DO was decreased from normoxia (>95% air saturation) to 10% air saturation within 120 min and maintained at 10% for 100 min by bubbling in nitrogen. Afterwards, DO was increased to 95% within 120 min by bubbling in air. Data from a total of seven trials is shown here (average temperature: 24.9 ± 0.5 °C).

Desired values of 10% and 95% air saturation were reached within 120 minutes and DO trajectories were highly correlated across trials (Pearson's R >= 0.999, Fig. S2C). However, DO curves did show a step-pattern with partial overshoots, especially during DO reduction. During maintenance of 10% air saturation the PID control produced slightly more hypoxic conditions than desired (9.67 ± 0.47% air saturation), normoxic values were reached with higher accuracy (95.06 ± 0.19% air saturation). Overall, this experiment is an example for a relatively simple application of DO control. The water surface-volume ratio was small, which reduced undesired influx of atmospheric oxygen. The tank was not compartmentalized, which was beneficial for water mixing and resulted in low latencies between adjustment of gas flow and changes of DO. Thus, the system could measure DO and adjust gas flow with a relatively high sample rate of 0.5 Hz, which improves the performance of the PID control algorithm. The motorized needle valve created a more continuous gas flow than e.g., when using a solenoid valve. This is beneficial when the fish might be disturbed by sudden intervals of nitrogen bubbling. We used a shaft coupling to connect a stepper motor to a manual needle valve and thus created a cheap motorized valve which worked reliably after all components and the rotational position of the motor shaft were fixed. The magnitude of overshoots during change of DO was reduced by adjusting the tunings of the PID control algorithm and reducing nitrogen pressure at the outlet.

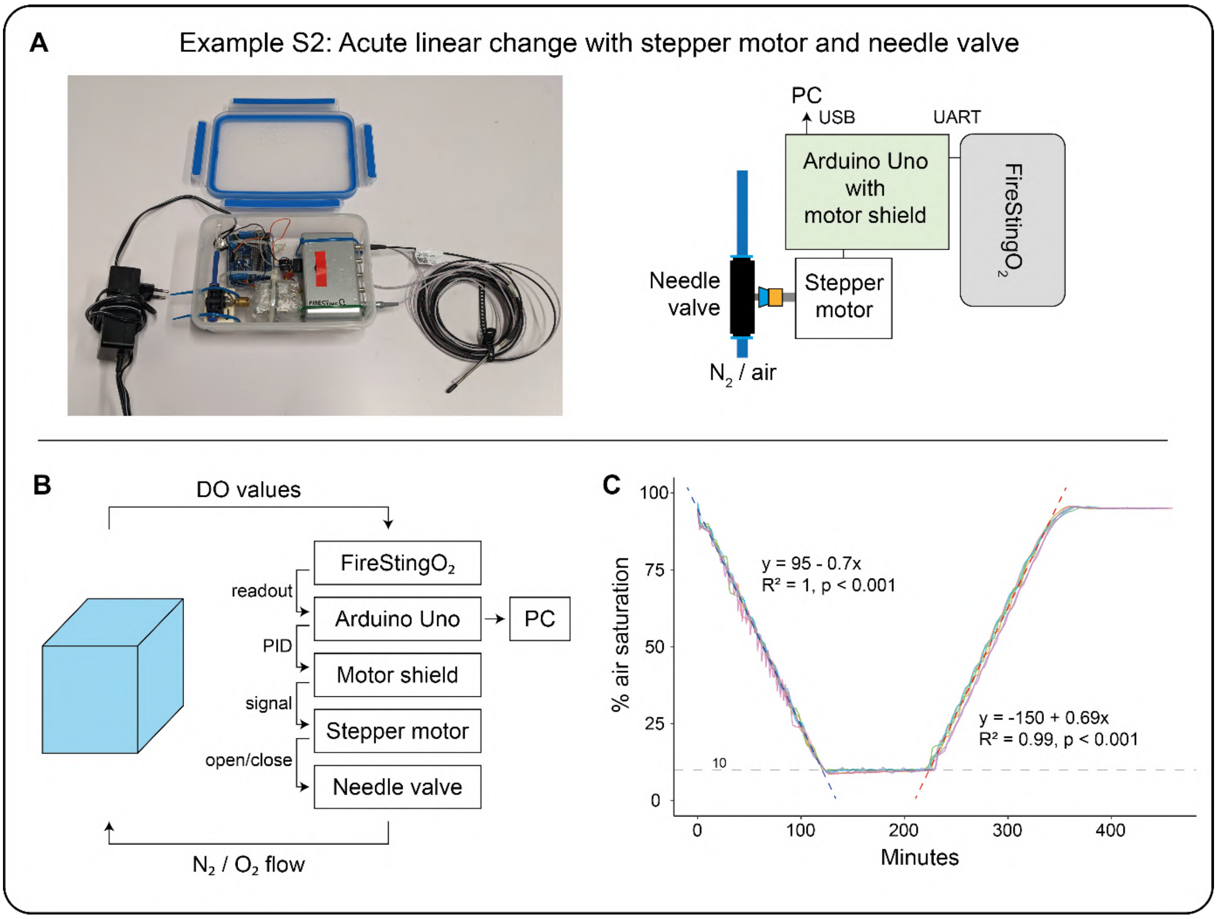

Fig. S2. A DO control system using a motorized needle valve. A: Components of the system, housed in a plastic box (left), and as schematic representation. B: Measurement principle. C: Measurement results. Pearson's correlation coefficients are shown for the linear change periods of the trial. Dashed horizontal lines show target DO setpoints. PID: proportional-integral-differential controller.

Example S3: Stepwise DO Change in a Shuttle-Box Tank using a Mass-Flow Controller

In this experiment, DO was decreased in a stepwise sequence in a shuttle-box tank (Loligo Systems, Viborg, Denmark). The shuttle-box consisted of two round compartments with a diameter of 50cm each, that were connected through a passage (width: 7.5cm, length: 10cm). In each compartment, water was exchanged with a pump (Universalpumpe 1046, Eheim) between the compartment and a buffer tank in which a gas diffusor was placed to bubble nitrogen into the water. A total water volume of 60L was used. Plexiglass lids were placed on the water surface in the compartments of the shuttle-box to reduce the diffusion of atmospheric oxygen into the water. The DO control system consisted of an Arduino Uno R3 that was connected to a FireStingO_2 sensor via serial connection (Fig. S1, 2B). Per side of the shuttle-box, one robust DO probe was placed in the round compartment and one robust DO probe was placed in the corresponding buffer tank. The temperature probe was placed in the passage between the compartments of the shuttle-box. In the buffer tanks, DO responded with a short latency to gas input. However, because the water exchange rate between the buffer tanks and the round compartments was limited, the latency between gas input and change of DO in the compartment was high, and DO values varied substantially between the buffer tanks and the compartments (Table S3). As a trade-off between accuracy and latency, we calculated a 1:2 weighted average of DO measured in the buffer tank and DO measured in the compartment. The weighted average was then compared to the set value and gas flow was adjusted accordingly. Temperature and DO were measured every ten seconds, values were sent from the Arduino to a PC via USB connection and visualized using SerialPlot (Fig. 3C). The gas flow was regulated through a mass-flow-controller (1259-V-10K-S, MKS Instruments Deutschland GmbH, München, Germany) that was powered by a +/-15V power supply and controlled via the analog output of the Arduino. Air saturation was lowered in one side of the shuttle-box to 70, 50, 30, 25, 20, 15 and 10% while normoxic conditions were maintained in the other side. Each air saturation value was maintained for 10 min and then changed to the next value within 10 min. Data from a total of 32 trials are shown here (average temperature: 23.4 ± 0.6°C).

DO values showed much higher variability than in experiment 1, although they were still highly correlated across trials (Pearson's R >= 0.987, Fig. 3D). The reduction to 15% air saturation was successful in 27 of 32 trials but 10% air saturation was not reached (14.13 ± 2.63% air saturation). Overall, DO control in the shuttle-box was much more challenging than in a simple tank. This was mainly due to three factors: large surface-volume ratio, poor water mixing between compartment and buffer tank, and undesired water mixing between hypoxic and normoxic compartments of the shuttle-box. We minimized influx of atmospheric oxygen by covering open water surfaces with lids. Further, we elevated the shuttle-box relative to the buffer tanks to raise the water level (i.e. the proportion of total water volume) in the buffer tanks where the surface-volume ratio was lower and DO could be adjusted more directly. Finally, we conducted regular maintenance of all components to ensure symmetrical flow rates in both compartments in order to reduce water exchange between the compartments.

Journal of Experimental Biology • Supplementary information

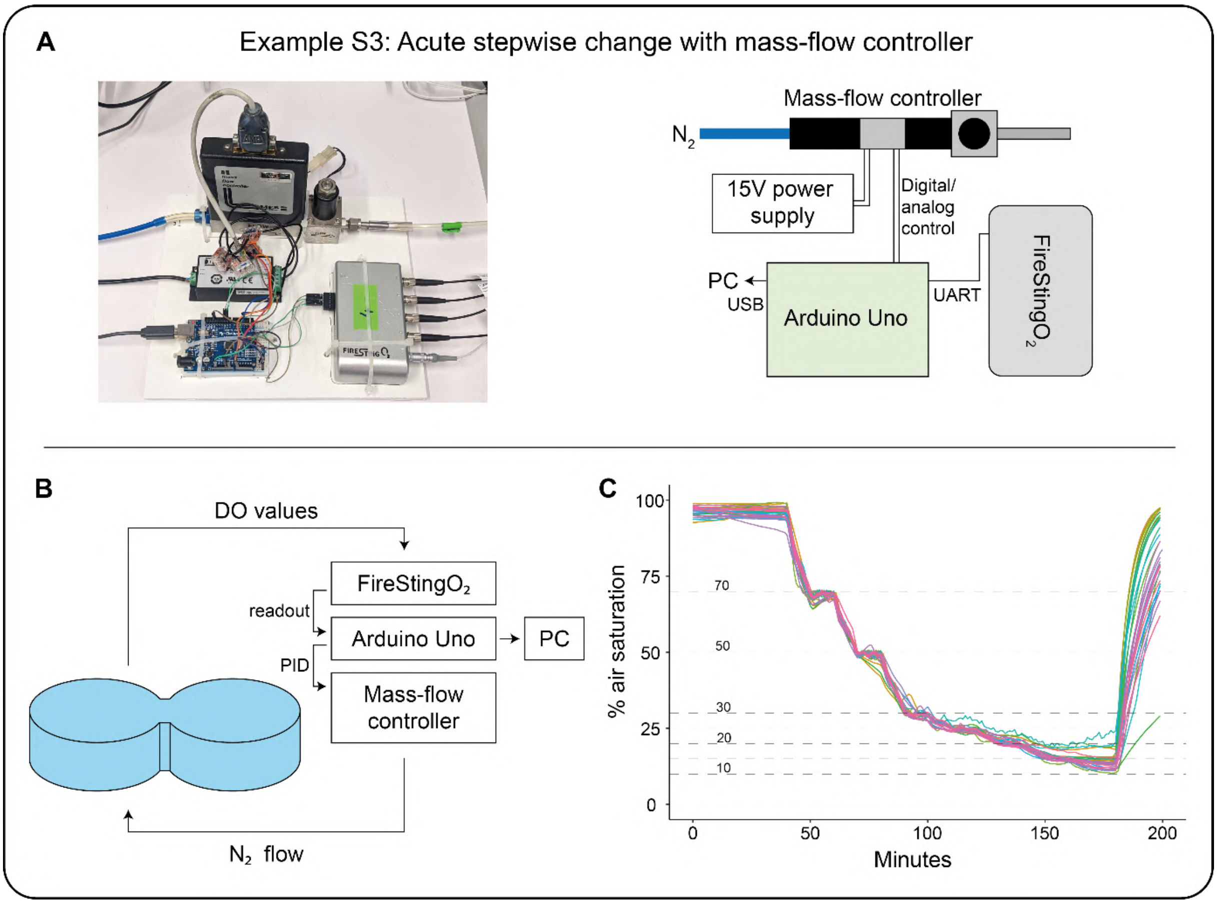

Fig. S3. A DO control system using a mass-flow controller for acute stepwise DO change in a shuttle-box. Dashed horizontal lines show target DO setpoints. PID: proportional-integral-differential controller.

Journal of Experimental Biology • Supplementary information

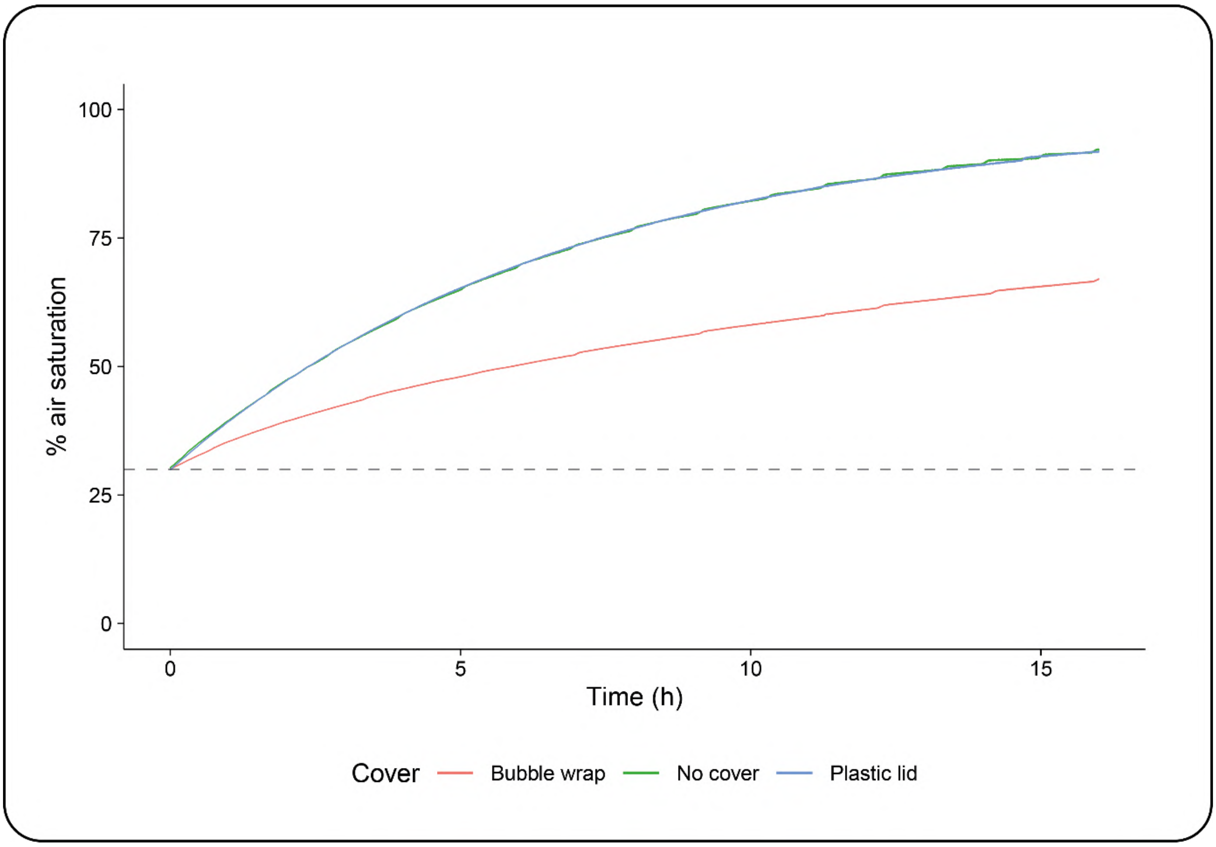

Fig. S4. Passive oxygenation in a tank (60 x 30 x 35 cm) with different cover methods. Air saturation was reduced to 30% (dashed line) and then measured for 16 hours without cover (green), with a plastic lid placed on top of the tank (blue), and with bubble wrap placed directly on the water surface (red). The water was mixed with two small pump and maintained at 25°C.

Table S1. Components of the oxygen control systems. Prices are estimated based on a survey of online shops (as of May 2024).

Reference	Component	Model	Price (USD)	Supplier examples
All Examples	DO meter	FireStingO2 (FSO2-C4), PyroScience GmbH, Aachen, Germany	4480	https://www.pyroscience.com/
	DO sensor	Robust Oxygen Probe (OXROB10), PyroScience GmbH, Aachen, Germany	380	https://www.pyroscience.com/
	Temperature sensor	Pt100 Temperature Probe (TSUB21), PyroScience GmbH, Aachen, Germany	180	https://www.pyroscience.com/
	7-Pin connector	PTSM 0,5/ 7-P-2,5, Art. No. 1778887, Phoenix Contact GmbH, Blomberg, Germany	4	https://www.digikey.com/ (global), https://www.rs-online.com/ (global)
	Cables and supplies	Generic	20	https://www.conrad.com/ (global), https://www.digikey.com/ (global)
Gas delivery	Gas diffusor	Aquarium Nano Bubble Stone 60mm, Boxtech, Shenzhen Bote Pet Equipment Co Ltd, Shenzen, China	14	https://www.amazon.com/
	Gas tubing	Generic (6 mm outer diameter pneumatic hose)	-	https://www.conrad.com/ (global), https://www.rs-online.com/ (global)
	Nitrogen	Alphagaz 1, Air Liquide S.A., Paris, France	-	https://www.airliquide.com/
Examples 1-2, S1	Microcontroller	Arduino Uno, Arduino LLC, Monza, Italy	25	https://www.conrad.com/ (global), https://www.digikey.com/ (global)
	Relay board	TC-9072472 2 Relay Module, Tru Components, Wels, Austria	8	https://www.conrad.com/ (global), https://www.amazon.com/
	Solenoid valve	Pro Valve 621.06.04131.12VDC, Provalve Armaturen GmbH, Niederndodeleben, Germany	40	https://www.conrad.com/ (global)
	Valve adapter plug	Precon B-12443-0127 12 - 24 V/DC, Nass Magnet, Germany	10	https://www.conrad.com/ (global)
	12V power supply	Generic (VC-11320935, Voltcraft, Germany)	15	https://www.conrad.com/ (global), https://www.digikey.com/ (global)
	Water pumps	Generic (CompactON300, Eheim, Deizisau, Germany)	20	Aquarium supply
	Heating rod	Generic (Thermocool 200, Eheim, Deizisau, Germany)	25	Aquarium supply
	Thermostat	ITC-308, Inkbird, Shenzen, China	30	https://www.amazon.com/
Example 3	Microcontroller	Arduino Mega 2560, Arduino LLC, Monza, Italy	50	https://www.conrad.com/ (global), https://www.digikey.com/ (global)
	Display	LCD Shield kit, Adafruit Industries LLC, New York, USA	25	https://www.digikey.com/ (global), https://www.mouser.com/ (global)

Journal of Experimental Biology • Supplementary information

	SD card logging	Data Logger Shield, Adafruit Industries LLC, New York, USA	15	https://www.digikey.com/ (global), https://www.mouser.com/ (global)
	4 relay board	TC-9927216, Tru Components, Wels, Austria	13	https://www.conrad.com/ (global), https://www.amazon.com/
	4 solenoid valves	6011 24V (art. 163521), Bürkert GmbH, Ingelfingen, Deutschland	240	https://www.burkert.com/ (global)
	5V power supply	Generic (VC-11258705, Voltcraft, Germany)	20	https://www.conrad.com/ (global), https://www.digikey.com/ (global)
	9V power supply	Generic (VC-11258705, Voltcraft, Germany)	20	https://www.conrad.com/ (global), https://www.digikey.com/ (global)
	24V power supply	Generic (OWA-60E-24, Mean Well, New Taipei City, Taiwan)	30	https://www.conrad.com/ (global), https://www.digikey.com/ (global)
	Housing	Generic (2007125K, OBO Bettermann, Menden, Germany)	30	https://www.conrad.com/ (global), https://www.amazon.com/
Electrochemical DO sensor circuits	DO sensor and signal conversion circuit	Surveyor Analog Dissolved Oxygen Kit (#Kit-105DX), AtlasScientific LLC, New York, USA	175	https://atlas-scientific.com/kits/surveyor-analog-do-kit/
	DO sensor and signal conversion circuit	Gravity Analog Dissolved Oxygen Sensor Kit (SEN0237-A), DFRobot, Shanghai, China	170	https://www.dfrobot.com/product-1628.html
Example S2	Microcontroller	Arduino Uno, Arduino LLC, Monza, Italy	25	https://www.conrad.com/ (global), https://www.digikey.com/ (global)
	Motor shield	Motor Shield V2, Adafruit Industries LLC, New York, USA	20	https://www.conrad.com/ (global), https://www.digikey.com/ (global)
	Stepper motor	NEMA-17 12V 350mA, Adafruit Industries LLC, New York, USA	15	https://eu.robotshop.com/ (EU, US), https://www.digikey.com/ (global)
	Shaft coupling	Generic	5	https://www.conrad.com/ (global), https://www.omc-stepperonline.com/
	Flow regulator valve	Generic (Shuny 6mm push-in valve)	10	https://www.conrad.com/ (global), https://www.rs-online.com/ (global)
	12V power supply	Generic (VC-11320935, Voltcraft, Germany)	15	https://www.conrad.com/ (global), https://www.digikey.com/ (global)
Example S3	Microcontroller	Arduino Uno, Arduino LLC, Monza, Italy	25	https://www.conrad.com/ (global), https://www.digikey.com/ (global)
	Mass-flow controller	1259-V-10K-S, MKS Instruments Deutschland GmbH, München, Germany	450	https://www.labexchange.com/
	15V Power supply	ECL30UD02-S, XP Power, Singapore, China	72	https://www.digikey.com/ (global), https://www.mouser.com/ (global)

Journal of Experimental Biology • Supplementary information

Table S2. Core functions of the Ardoxy library.

Function	Purpose	Arguments	Returns
begin()	Establish connection with FireStingO2 sensor	-	-
end()	Close connection with FireStingO2 sensor	-	-
measure()	Send user defined measurement command to the meter	Command as character string	1 – success<br>0 – no connection<br>9 – mismatch
measureSeq()	Measure a sequence of DO and temperature on a channel	Measurement channel as integer	1 – success<br>0 – no connection<br>9 – mismatch
measureDO()	Measure DO on a channel	Measurement channel as integer	1 – success<br>0 – no connection<br>9 – mismatch
measureTemp()	Measure temperature	-	1 – success<br>0 – no connection<br>9 – mismatch
readout()	Send user defined readout command to the meter	Command as character string	Measured value<br>0 – mismatch
readoutDO()	Read out DO measurement result	Measurement channel as integer	Measured value<br>0 – mismatch
readoutTemp()	Read out temperature measurement result	Measurement channel as integer	Measured value<br>0 – mismatch

Table S3. Dissolved oxygen (DO) measured at different setpoints in the buffer tank and one side of the shuttle-box. Values are given as % air saturation of one example trial, averaged over the 10-minute period over which stable DO values were held during the trial. The weighted average was calculated as (1 x [Buffer tank] + 2 x [Shuttle-box])/3 and was used to approximate the DO in the shuttle-box and to control the nitrogen gas output.

Setpoint	Buffer tank	Shuttle-box	1:2 weighted average
70	65.3 ± 3.3	70.0 ± 0.8	68.4 ± 1.2
50	45.8 ± 2.8	51.2 ± 0.8	49.4 ± 0.9
30	24.8 ± 1.6	32.2 ± 1.1	29.7 ± 0.6
25	20.7 ± 0.1	26.5 ± 0.5	24.6 ± 0.3
20	15.6 ± 0.4	22.4 ± 0.2	20.1 ± 0.2
15	10.3 ± 0.6	18.3 ± 0.4	15.6 ± 0.4
10	9.6 ± 0.0	17.3 ± 0.1	14.7 ± 0.1

Journal of Experimental Biology • Supplementary information

Table S4. List of example studies that have reported long-term DO data (values are mean ± standard deviation except for Reardon and Chapman 2010, where values are reported with standard error, SE).

**Reference**	**Duration**	**Air saturation**
Ackerly et al. 2023	6 weeks<br>8 days	33.3 ± 0.2%<br>31.3 ± 0.3%
Ackerly et al. 2018	8 weeks	17.5 ± 3.5%
Pan et al. 2017	3 weeks	31 ± 3.5%
Reardon and Chapman 2010	6 months	16.9 ± 0.1 (SE)%
This study	8 weeks	14.96 ± 0.38%

References

Ackerly KL, Krahe R, Sanford CP, Chapman LJ (2018) Effects of hypoxia on swimming and sensing in a weakly electric fish. J Exp Biol 221. https://doi.org/10.1242/jeb.172130

Ackerly KL, Negrete B, Dichiera AM, Esbaugh AJ (2023) Hypoxia acclimation improves mitochondrial efficiency in the aerobic swimming muscle of red drum (*Sciaenops ocellatus*). Comp Biochem Physiol A Mol Integr Physiol 282:111443. https://doi.org/10.1016/j.cbpa.2023.111443

Pan YK, Ern R, Morrison PR, Brauner CJ, Esbaugh AJ (2017) Acclimation to prolonged hypoxia alters hemoglobin isoform expression and increases hemoglobin oxygen affinity and aerobic performance in a marine fish. Sci Rep 7:7834. https://doi.org/10.1038/s41598-017-07696-6

Reardon EE, Chapman LJ (2010) Hypoxia and energetics of mouth brooding: is parental care a costly affair? Comp Biochem Physiol A Mol Integr Physiol 156:400–406. https://doi.org/10.1016/j.cbpa.2010.03.007

Journal of Experimental Biology • Supplementary information
